# Molecular Biogeography of Tribe Thermopsideae (Leguminosae): A Madrean-Tethyan Disjunction Pattern with an African Origin of Core Genistoides

**DOI:** 10.1155/2015/864804

**Published:** 2015-05-31

**Authors:** Ming-Li Zhang, Jian-Feng Huang, Stewart C. Sanderson, Ping Yan, Yu-Hu Wu, Bo-Rong Pan

**Affiliations:** ^1^Key Laboratory of Biogeography and Bioresource in Arid Land, Xinjiang Institute of Ecology and Geography, Chinese Academy of Sciences, Urumqi, Xinjiang 830011, China; ^2^Institute of Botany, Chinese Academy of Sciences, Beijing 100093, China; ^3^Xishuangbanna Tropical Botanical Garden, Chinese Academy of Sciences, Kunming, Yunnan 650223, China; ^4^Shrub Sciences Laboratory, Intermountain Research Station, Forest Service, U.S. Department of Agriculture, UT 84601, USA; ^5^School of Life Science, Shihezi University, Shihezi, Xinjiang 832003, China; ^6^Northwest Plateau Institute of Biology, Chinese Academy of Sciences, Xining, Qinghai 810001, China

## Abstract

Thermopsideae has 45 species and exhibits a series of interesting biogeographical distribution patterns, such as Madrean-Tethyan disjunction and East Asia-North America disjunction, with a center of endemism in the Qinghai-Xizang Plateau (QTP) and Central Asia. Phylogenetic analysis in this paper employed maximum likelihood using ITS, *rps*16, *psb*A-*trn*H, and *trn*L-F sequence data; biogeographical approaches included BEAST molecular dating and Bayesian dispersal and vicariance analysis (S-DIVA). The results indicate that the core genistoides most likely originated in Africa during the Eocene to Oligocene, ca. 55-30 Ma, and dispersed eastward to Central Asia at ca. 33.47 Ma. The origin of Thermopsideae is inferred as Central Asian and dated to ca. 28.81 Ma. *Ammopiptanthus* is revealed to be a relic. Birth of the ancestor of Thermopsideae coincided with shrinkage of the Paratethys Sea at ca. 30 Ma in the Oligocene. The Himalayan motion of QTP uplift of ca. 20 Ma most likely drove the diversification between Central Asia and North America. Divergences in East Asia, Central Asia, the Mediterranean, and so forth, within Eurasia, except for *Ammopiptanthus*, are shown to be dispersals from the QTP. The onset of adaptive radiation at the center of the tribe, with diversification of most species in *Thermopsis* and *Piptanthus* at ca. 4-0.85 Ma in Tibet and adjacent regions, seems to have resulted from intense northern QTP uplift during the latter Miocene to Pleistocene.

## 1. Introduction

In the Leguminosae, the so-called core genistoides includes tribes Crotalarieae, Genisteae, Podalyrieae, Thermopsideae, Euchresteae, and Sophoreae sensu strictu [1–8]. Tribe Thermopsideae includes seven genera and about (43)-45-(46) species and occurs in the temperate regions of Eurasia and North America [6, 9]. Of them,* Pickeringia*, with one species endemic to western North America, has been transformed into* Cladrastis–Styphnolobium* [5, 10].* Thermopsis* and* Baptisia *are two perennial herbaceous genera, respectively, in distributions of an East Asian-North American disjunction and North American endemism.* Anagyris, Piptanthus*, and* Ammopiptanthus* are shrubby and in Eurasia.* Anagyris* includes two species and occurs around the Mediterranean Basin [11, 12].* Piptanthus *and* Ammopiptanthus* mainly occur in China, the former in Sino-Himalayan [13] and the latter in Central Asian regions [14]. New monotypic genus* Vuralia* recently is segregated from* Thermopsis* in Turkey [15].

Molecular evidence above the rank of genus has provided a foundation for Thermopsideae systematics and biogeography [4, 5, 16–19]. However, due to a lack of sufficient species sampling, a dense addition of species at generic level is necessary. Wang et al. [14] carried out a comprehensive systematic study of Thermopsideae on the basis of dense species addition and ITS sequences. Biogeographically, the fossil record indicates that the three legume subfamilies appeared in the early Eocene, and extensive diversification and origin of most of the woody legume lineages occurred in the middle Eocene [20]. Schrire et al. [21] divided the distribution patterns of ca. 730 legume genera into four biomes, that is, succulent, grass, rainforest, and temperate, with temperate groups possessing the largest numbers. From macrofossils of leaves and pods, the origin of legumes appears unlikely to have been much before 60 Ma, and, from that time, a rapid diversification among major clades took place [22]. In contrast with a proposed West Gondwana origin of the family [23, 24] or a “moist equatorial megathermal” origin, recent studies favor an origin in the seasonally dry to arid tropical Tethyan seaway corridor [21]. Lavin et al. [22] established a comprehensive schematic chronogram of legumes based on sequence data and fossil constraint, employing a total of 324 species. However, only three species of Thermopsideae were sampled. Lavin et al. [22] estimated ca. 26.5 Ma for the time of origin of Thermopsideae, and Ortega-Olivencia and Catalán [12] dated the appearance of* Anagyris* to late Miocene (8.2 ± 4.5 Ma). Xie and Yang [25] estimated* Ammopiptanthus* to have originated in early Miocene ca. 20-21 Ma.

Exceptionally, in the Wang et al. [14] study, although there was strong support for the tribal clade, the systematic position of* Ammopiptanthus* was suspected as not being a member of Thermopsideae because of the nesting of three* Sophora* species with it, resulting in* Ammopiptanthus* being placed in a basally branching position with respect to the rest of the tribe. Many studies have speculated that* Piptanthus, Ammopiptanthus, Thermopsis*, and so forth in the tribe originated in the Tertiary [14, 26–29], but the exact time and place of origin have remained poorly understood.

In summary, Thermopsideae contains many attractive biogeographical topics, Central Asia, East Asia, and QTP endemism and Madrean-Tethyan disjunction, East Asia-North America disjunction, Tertiary origin, and so forth. Therefore, this paper attempts to reconstruct the phylogeny of the tribe using four genes and, afterward, focuses on the tribe Thermopsideae biogeography by employing biogeographical molecular dating and S-DIVA approaches to explore the spatiotemporal origin and evolution of Thermopsideae and its evolutionary dynamics; to confirm the Madrean-Tethyan disjunction using Thermopsideae; and to discuss the East Asia-North America disjunction, Central Asian endemism, QTP endemism, and so forth.

## 2. Materials and Methods

### 2.1. Taxon Sampling

We sampled 32 individuals of 20 species, mainly from China, belonging to three genera,* Thermopsis*,* Piptanthus*, and* Ammopiptanthus* of Thermopsideae; see Table 1. Outgroups were selected from* Sophora* (*S. davidii, S. flavescens, *and* S. microphylla*),* Podalyria* (Podalyrieae), and* Cytisus* (Genisteae); see Supplementary Material S 1 available online at http://dx.doi.org/10.1155/2015/864804. More outgroup species were used in ITS phylogeny; also see S 2.

### 2.2. DNA Sequencing

Total genomic DNA was extracted using the CTAB method [30]. The polymerase chain reaction (PCR) was used for amplification of double stranded DNA. A 25 *μ*L reaction system contained 0.25 *μ*L of Ex Taq, 2.5 *μ*L of 10× Ex Taq buffer (Mg^2+^ concentration of 25 mM), 2.0 *μ*L of dNTP mix (2.5 mM concentration for each dNTP), 1 *μ*L of the forward and reverse primers at 5 *μ*mol/*μ*L, and 0.5 *μ*L of template DNA. The following primers were used: for ITS, ITS1-F (5′-AGA AGT CGT AAC AAG GTT TCC GTA GC-3′) and ITS4-R (5′-TCC TCC GCT TAT TGA TAT GC-3′) [31], for trnL-F, trnLF (5′-CGA AAT CGG TAG ACG CTA CG-3′) and trnFR (5′-ATT TGA ACT GGT GAC ACG AG-3′) [32], for* psb*A-*trn*H,* psb*AF (5′-GTT ATG CAT GAA CGT AAT GCT C-3′) [33] and* trn*HR (5′-CGC GCA TGG ATT CAC AAT CC-3′) [34], and, for the intron of rps16,* rps*16F (5′-GTG GTA GAA AGC AAC GTG CGA CTT-3′), and for* rps*16R (5′-TCG GGA TCG AAC ATC AAT TGC AAC-3′) [35].

PCR amplifications were carried out using the following procedures: there was predenaturation at 94°C for 3 min., followed by 30 cycles of (1) denaturation at 94°C for 30 s, (2) annealing at 48°C–54°C for 30 s, and (3) extension at 72°C for 1 min.; at the end of these cycles, there was a final extension at 72°C for 10 min. PCR products were purified using the PEG precipitation procedure [36]. Sequencing reactions were performed by a company specializing in the procedure (Beijing Sanbo Biological Engineering Technology and Service Corporation, China). Sequences were aligned using CLUSTAL X software [37] and then adjusted by hand in BioEdit ver. 5.0.9 [38].

### 2.3. Phylogenetic Analyses

Two datasets consisting of ITS and the 4-gene sequences combined (ITS+3cpDNA) were prepared for phylogenetic analysis. The 4-gene dataset was examined using the incongruence length difference (ILD) tests [39], implemented in PAUP version 4.0b10 [40], with 100 homogeneity replicates, 10 random addition sequences, tree-bisection-reconnection (TBR) branch swapping on best only, and MULTREES on, and this was performed to test whether the four datasets could be combined. The data partitions of four genes were not significantly incongruent on the basis of the ILD tests (*P* = 0.01).

Phylogenetic analysis by Maximum Likelihood (ML) of the 4-gene combined sequences was conducted using PAUP ^*^ 4.0b10 [40].

For ML analysis, the best fitting DNA substitution model was found employing Modeltest 3.6 [41], of which the Akaike information criterion (AIC) was selected on the basis of the log likelihood scores of 56 models [41]. For the dataset, the TrN+G model was selected as the most appropriate in Modeltest 3.5, with the nucleotide frequencies A = 0.3283, C = 0.1676, G = 0.1985, T = 0.3055, the shape parameter = 0.6264, and an assumed proportion of invariable (PIV) sites = 0. Clade support for the phylogenetic tree was estimated, employing bootstrap values in PAUP and posterior probability values in MrBayes software.

In order to obtain comprehensive molecular dating, ITS sequence data covered broad outgroups including the four core genistoides tribes and seven fossil genera, which came from our data and from GenBank; see S 2. The final dataset comprised 107 species and 722 bps.

### 2.4. Estimating Divergence Times

#### 2.4.1. Fossil Constraints

Legumes have rich fossil records [20, 42], but there are fewer fossils assignable to the core genistoides and Thermopsideae. Most fossils of legume genera date to the Miocene, with several approaching the Eocene and Paleocene, with* Bauhinia* and* Cercis* extending even to the Late Cretaceous [42, 43]. Seven fossil genera are used as outgroups; see Table 2. The occurrence of* Sophora* in the Oligocene-Miocene is credible, since its fossils are known from the Eocene of eastern Siberia and North America [42, 43]. In China,* Sophora* fossils have been found in Oligocene strata from Heilongjiang province, the Miocene from Shandong and Yunnan provinces, and Pliocene from Shanxi province [42, 43].* Acacia* in the Eocene is also well represented in museum collections.* Dalbergia* fossils, including leaves and fruit, have been recorded at the Eocene-Miocene boundary in North America, the Oligocene-Miocene boundary in Europe, and in the Miocene in Yunnan Province, China. Fossil leaves of* Pueraria* appeared in the Miocene in Shandong and Yunnan provinces, China [42, 43], and* Cladrastis* has been dated to middle Eocene [22].

In terms of the ancient fossil record and the phylogenetic tree, the root taxon was considered as* Cercis*. In the southern China Guangdong province,* Cercis* fossils haves been found from the Late Cretaceous to Eocene, in the Oligocene of Yunnan province, and the Miocene of Shandong and Qinghai provinces. Therefore, this genus is regarded as the root taxon and the age of its ancestor is constrained as 60 Ma. This root constraint is in agreement with Lavin et al. [22], by whom the ancestor of* Polygala* and* Cercis* was constrained at 60 Ma. Detailed information are described in Table 2.

The outgroup fossil dates were used as the constraint minimum ages; that is, the maximum fossil dates were selected as the generic minimum age of the most recent common ancestor (MRCA).

#### 2.4.2. Dating Implementation

Currently, phylogenetic dating approaches include r8s, PAML, and BEAST. Of them, BEAST has an advantage for practical applications because of its nondependence on a phylogenetic tree, and convenient implementation software (BEAST v1.46, http://beast.bio.ed.ac.uk). Moreover, a relaxed molecular clock and Bayesian MCMC search optima are available within it [44, 45].

BEAST was implemented [46] using a Yule process speciation prior to an uncorrelated lognormal model of rate variation and a normal distribution. Tracer v1.4 was used to measure the effective sample size of each parameter and mean and 95% credibility intervals. Two separate MCMC analyses were run for 20,000,000 generations and sampled every 1000 generations. After discarding as burn-in the first 10% of trees searched, the mean and 95% credibility intervals of MRCA nodes were calculated by TreeAnnotator v1.4.8. and visualized by FigTree v.1.2.4 [46].

### 2.5. Biogeographic S-DIVA

DIVA is used to infer mainly ancestral distributions and biogeographical events [47]; it is an event-based method that optimizes ancestral distributions by assuming a vicariance explanation, while incorporating the potential contributions of dispersal and extinction, despite minimizing these under a parsimony criterion [47, 48]. Nylander et al. [49] proposed a modified approach to DIVA naming it Bayes-DIVA because it integrates biogeographical reconstructions of DIVA over the posterior distribution of a Bayesian MCMC sample of tree topologies. Bayes-DIVA is also referred to as S-DIVA [50].

The BEAST dating tree (Figure 2) was treated as a fully resolved phylogram for use as a basis for S-DIVA, and 791 post burnin trees derived from the BEAST analysis were used for ancestral area reconstruction in the program S-DIVA beta version 1.9. S-DIVA was performed with constraints of maximum areas 2 at each node, to infer possible ancestral areas and potential vicariance and dispersal events.

Geographic areas were chosen to cover the distributions of the four core genistoides tribes, especially tribe Thermopsideae. Seven geographic endemic areas were defined in this study: East Asia, Central Asia, the Mediterranean, Africa, Russia (including Central East, Caucasus, and northeastern Russia), North America, and Tibet. Because of its species richness and endemism, the QTP, Tibet is regarded as an area separated from the East Asian floristic region [13].

## 3. Results

### 3.1. Phylogenetic Analyses

#### 3.1.1. 4-Gene Combined Analysis

**Figure 1:**
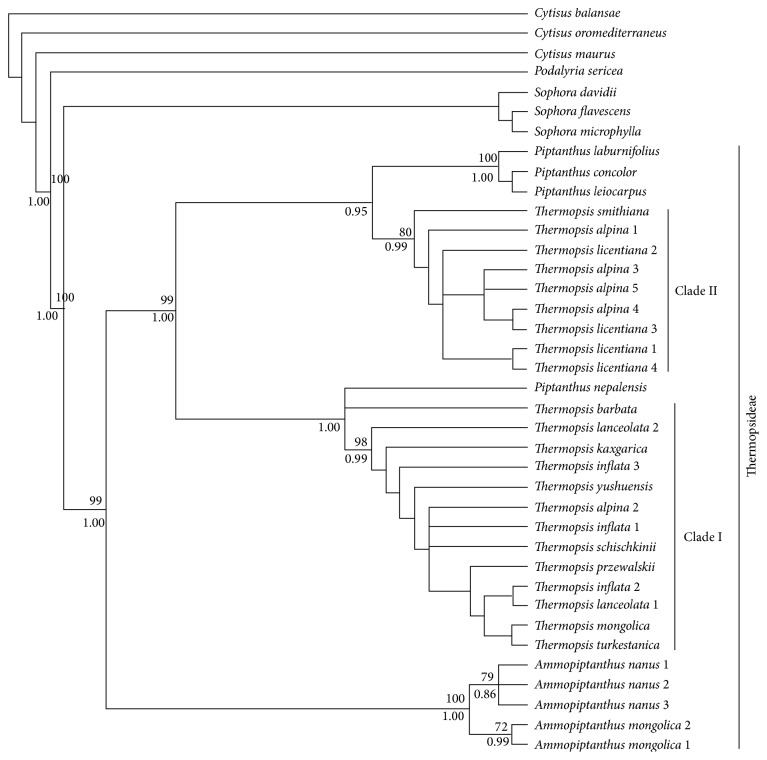
Phylogenetic tree resulted from maximum likelihood analysis of the combined dataset of 4 genes (ITS,* trn*L-F,* psb*A-*trn*H, and* rps*16). Bootstrap support values > 50% above branches and posterior probability support > 0.5 below branches are indicated.

The 4-gene combined dataset included 38 samples and 3099 bps; 496 variable characters were parsimony-uninformative and 421 were parsimony-informative. ML analysis resulted in three optimum trees, topologically almost equivalent; one of them is shown in Figure 1. Bootstrap support from PAUP and Bayesian posterior probability are labeled on the nodes in Figure 1.

Thermopsideae and* Ammopiptanthus*, respectively, formed a monophyletic group with high support, near 100% bootstrap (BT) and posterior probability values (PP).* Piptanthus* did not form a monophyletic group, since* P. nepalense* was placed outside of the genus (Figure 1). Even though the samples of* Thermopsis* came only from China (Figure 1), the results show that section* Thermopsis* sensu Sa et al. [28] can apparently be divided into two clades (Figure 1).

#### 3.1.2. ITS Analysis

**Figure 2:**
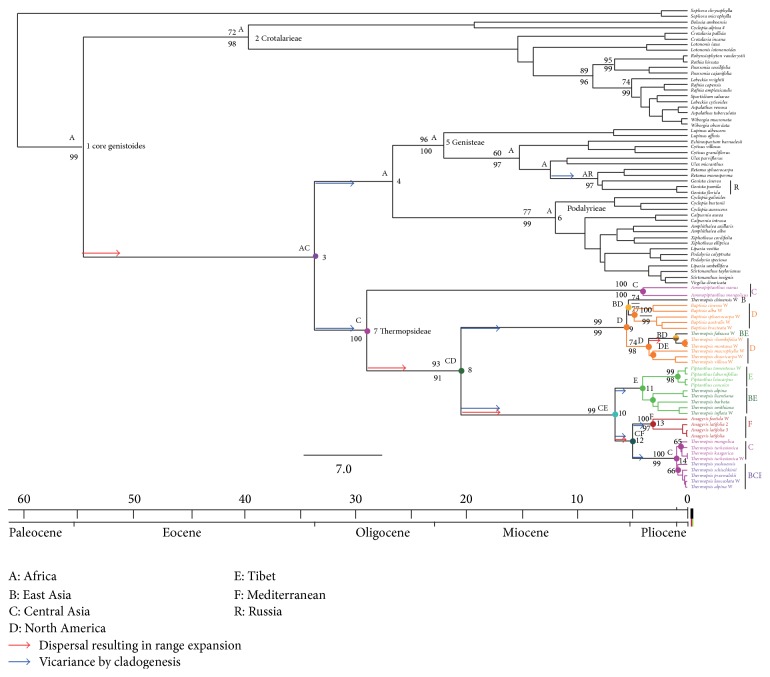
Chronogram of relaxed Bayesian BEAST on the basis of the ITS dataset. Estimated times (Ma) with 95% HPD credibility intervals at concerned nodes were 1 : 54.43 (53.04–58.85), 2 : 39.45 (16.98–54.54), 3 : 33.47, 4 : 26.51 (13.59–42.87), 5 : 21.91 (7.09–23.65), 6 : 11.89 (4.78–15.64), 7 : 28.81 (10.95–41.02), 8 : 20.32 (6.9–22.98), 9 : 5.48 (2.99–10.81), 10 : 6.5 (3.38–11.11), 11 : 4, 12 : 4.9 (1.6–6.89), 13 : 3.08 (0.26–3.2), and 14 : 0.97 (0.46–2.8). Bootstrap support values > 50% above branches and posterior probability values > 0.5 below branches are indicated. “W” behind species name means species with the sequence data come from GenBank produced by Wang et al. [14]. S-DIVA optimal reconstruction of hypothesized ancestral areas at nodes and 10 dispersals with vertical line on branches are illustrated.

The ITS BEAST implementation yielded a phylogenetic tree and dating chronogram; see Figure 2. This topology of tree is in rough agreement with that of the previous ITS tree [14] and our 4-gene tree (Figure 1). It indicates that* Ammopiptanthus* and Thermopsideae are monophyletic groups, respectively. Importantly, this chronogram has a temporal evolutionary significance for the Thermopsideae, Podalyrieae, Crotalarieae, Genisteae, and so forth.

In contrast with previous phylogenies, especially Wang et al. [14], this ITS tree places* Ammopiptanthus* within Thermopsideae rather than outside of the tribe, and this is the same as in our 4-gene tree (Figure 1). The topological structure of the four tribes of core genistoides is also somewhat different from previous studies [19]; Thermopsideae is related to the cluster of Genisteae and Podalyrieae, while Crotalarieae is more isolated.

#### 3.1.3. Estimating Divergence Times

Using seven fossil genera as constraints and outgroups, for 107 species and ITS dataset, the estimated root age of the four tribes of core genistoides was ca. 54.43 Ma and that of Thermopsideae was ca. 28.81 Ma, as presented in Figure 2. The estimated crown ages of the four tribes range from later Eocene 39.45 Ma (Crotalarieae) to Miocene 11.89 Ma (Podalyrieae).

Within Thermopsideae, five genera are well monophyletic, respectively, with credible crown and stem ages excluding* Thermopsis*.* Ammopiptanthus* has stem age ca. 28.81 Ma, namely, crown age of Thermopsideae. In order to discuss the origin and evolution of taxa, a geological scale was appended to the BEAST diagram in Figure 2.

#### 3.1.4. Biogeographic S-DIVA

**Figure 3:**
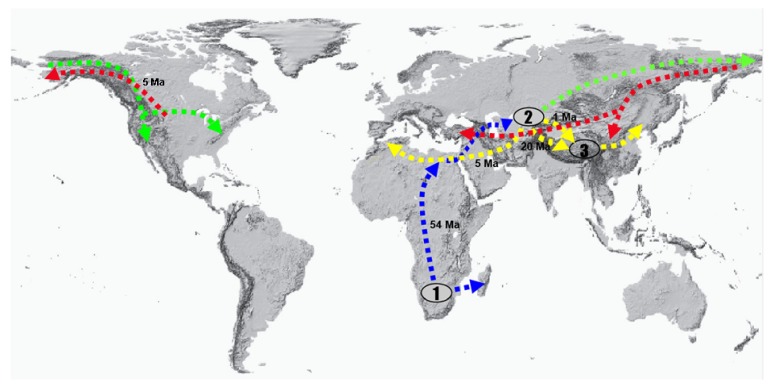
Scheme of dispersal routes from the biogeographical S-DIVA analysis, Figure 2. The blue dashed line indicates the origin center of core genistoides from Africa (elliptic 1), arriving in Central Asia (elliptic 2), which was the place of origin of the Thermopsideae; then there was a dispersal to North America. Routes are shown by green dashed lines. From Central Asia (elliptic 2), a dispersal westward via the Caucasus to the Mediterranean with genus* Anagyris* and dispersal and adaptive radiation to QTP (elliptic 3), from QTP to North China, are shown by yellow dashed lines. Dispersal from North America at about late Miocene eastward to East Asia and Mediterranean is shown by red lines.

Reconstruction of ancestral areas with S-DIVA (Figure 2) suggested that the ancestral distribution area of core genistoides is Africa (A) and that of Thermopsideae is possibly Central Asia (C) and that* Ammopiptanthus* is directly derived from Thermopsideae. Extant taxa of North America and the QTP are shown to be dispersals from Central Asia; several events of dispersal and vicariance are illustrated in Figure 2. The most distinct dispersal for the core genistoides is from Africa to Central Asia. From the QTP, a dispersal was westward via the Himalayas to the Mediterranean for the genus* Anagyris*, and dispersal and adaptive radiation to East Asia and Central Asia. The eastward line is via the Bering Strait to North America (Figures 2 and 3).

## 4. Discussion

### 4.1. Systematics of Thermopsideae

In the previous ITS phylogenetic tree [14], the five genera of Thermopsideae formed a well resolved monophyly, except for the fact that* Ammopiptanthus* fell outside of Thermopsideae due to the nesting of a few species of* Sophora*. In addition, diversification into East Asian and North American groups is observed in* Thermopsis*. From our 4-gene combined (Figure 1) and ITS trees (Figure 2), the monophyly of Thermopsideae and* Ammopiptanthus* is confirmed once more, and* Ammopiptanthus* is entirely included within Thermopsideae with high confidence support. Consequently, our enhanced species sampling and 4-gene tree (Figure 1) yield a distinct result compared with Wang et al. [14], mainly, that Thermopsideae is monophyletic, since the three* Sophora* species (*S. davidii, S. flavescens*, and* S. microphylla*) are out of the tribe; two phylogenetic clades are recognized, where the previous tree only had one (see Figure 1 of Wang et al. [14]; our ITS tree also has one clade see Figure 2). This probably will be useful for the revision of classification [28, 51, 52], especially for section* Thermopsis* sensu Sa et al. [28], of which most species occur in Asia. In addition, the previous taxonomic opinion of* Ammopiptanthus* being morphologically related to* Piptanthus* [26, 53] should be considered as reflecting a convergence, since our results (Figures 1 and 2) illustrate that they are separated in the tree.* Vuralia* has only one species* V. turcica* (Kit Tan et al.) and has a narrowed distribution (marshy side of Aksehir in Turkey) Uysal et al. [15], even though it had not been joined into the present dataset of Thermopsideae; however, together with* Thermopsis chinensis* and* Th. fabacea*, all are shown to be included into North America clade node 9 (Figure 2) [15].

### 4.2. Age and Distribution Pattern of Thermopsideae

The age of Thermopsideae has been estimated several times by the molecular dating approach. On the basis of fossil data, the genistoides crown node was constrained at 56.42 ± 0.2 Ma. Lavin et al. [22] dated Thermopsideae to ca. 26.5 Ma, but only three species were sampled, that is,* Piptanthus nepalense, Baptisia australis*, and* Thermopsis rhombifolia*. This node of the genistoides is placed at ca. 54.43 Ma (Figure 2), near this fossil constraint, confirming the validity of our dating. To estimate the age of* Anagyris*, Ortega-Olivencia and Catalán [12] employed numerous samples of the two species* A. foetida* and* A. latifolia* and added three other species in the tribe. Their results indicated that an estimated age of Thermopsideae was 27.2 ± 4.1 Ma and of* Anagyris* was 8.2 ± 4.5 Ma. The present paper dates Thermopsideae to ca. 28.81 Ma, which approaches the dates from previous studies by Lavin et al. [22] and Ortega-Olivencia and Catalán [12]. Therefore, the middle Oligocene ca. 28.81 Ma should be treated as the diversification age of the tribe.

Along with the significant global climate cooling and increased aridity from Eocene to Oligocene, seven distinctive biomes have been recognized for the Oligocene (38~24 Ma) [54]. At ca. 30 Ma, one of seven is the warm/cool temperate biome, with a wide band of broadleaved evergreen and deciduous woodland throughout central Eurasia and North America. This biome in its northernmost part just covers the distribution range of Thermopsideae. These woodlands and forests replaced the dominantly evergreen paratropical rainforest of the middle Paleocene and much of the Eocene [54]. Therefore, we can determine that the original accompanying vegetation of Thermopsideae was woodlands and forest, with broadleaved evergreen and deciduous plants. From the Oligocene ca. 30 Ma to middle-to-late Miocene, shrinkage of the Paratethys played an important role in causing transformation of the Central Asian climate from an oceanic to a continental condition [55]. As Hrbek and Meyer [56] have reviewed, the closing of the sea near the Oligocene/Miocene boundary had a major impact on the distribution of organism diversity. Therefore, origin and diversification of the Thermopsideae at ca. 30 Ma could therefore have been driven by the closing of the Paratethys, which resulted in a series of changes of environmental and ecological factors and profoundly affected the evolution of the tribe.

The Oligocene environment in Kazakhstan, with broad-leaved forest and swamps, was indicated to be a wet climate [57]. However, the Paleogene floristics of northwestern and central China evidenced by fossil data was dry and subtropical [58]. During the Oligocene, the climate of middle China is speculated to have been an arid/semiarid belt [59]. Therefore, climate in Oligocene Central Asia should have changed from western wet (relic locations of Paratethys shrinkage) in Kazakhstan to eastern dry in northwestern China. These wet environments and climates of western parts of Central Asia most likely fit the emergence of the ancestor of* Ammopiptanthus* and Thermopsideae, with a broad-leaved forest and a wet to arid climate.

Therefore, from these perspectives of time and place of origin, paleovegetation, and paleoclimate, we can confirm a balanced Oligocene Central Asian origin of Thermopsideae.

### 4.3. Central Asian Origin and Diversifications among Central Asia, the QTP, and North America

In terms of our inferences of dispersal and vicariance in Thermopsideae (Figures 2 and 3), we can speculate that, after origination in Central Asia, most of its broadleaved evergreen and deciduous ancestors probably soon became extinct. Only a few survived, a case being* Ammopiptanthus*, which evolved from the ancestor of Thermopsideae (Figure 2). From the Central Asian ancestor, Thermopsideae had a dispersal to North America at ca. 28.81 Ma and to the QTP at ca. 20.32 Ma (Figures 2 and 3).

As mentioned above, shrinkage of the Paratethys starting from the Oligocene [55] was a dynamic influence for Thermopsideae and most likely also drove the dispersal to North America (dispersal from node 7 Figures 2 and 3). The diversification age between Central Asia and QTP of ca. 20.32 Ma implies a response to QTP uplift and extension as a geological event. QTP uplift is presumed to have initiated very early but had a major phase near the Oligocene-Miocene boundary when loess deposition began and strengthened thereafter. The first phase (Gangdese motion ca. 40 Ma, 45~38 Ma) is characterized by the Indian plate subducting under the Eurasian plate, resulting in the rise of the Gangdise Mountains. High altitude conifers became abundant in the QTP starting at 38 Ma [60]. The second phase (Himalayan motion ca. 21 Ma, 25~17 Ma) is characterized by westward withdrawal of the Paratethys Sea and aridification of interior Asia. The rise or expansion of the QTP became sufficient for the initiation of dust deposition due to the Asian winter monsoon [59, 61, 62]. The third phase, uplift of northern and eastern parts of the QTP at many intervals during the late Neogene to Pleistocene [63–65] is correlated with appearance of ocean upwelling connected to development of the Asian summer monsoons. A particularly intense geologic uplift during this period was recorded in parts of the northernmost QTP at ca. 3.6 Ma [63, 66–68], which was accompanied by the intensification of monsoons to present levels.

These events strikingly influenced the ecology and environment of the QTP and adjacent regions, especially northern China. These ecological and environmental settings, consequently, can hold temporally the dispersal from Central Asia (see Figure 2, node 8) into the QTP.

The QTP and adjacent regions possess many endemic species in Thermopsideae (nodes 11,14 in Figure 2); these areas are shown by the analysis to be related mechanistically to Central Asia. A vicariance between the QTP and Central Asia is estimated at ca. 6.5 Ma (node 10, Figure 2), whereas those species of the QTP, North China, and Central Asia (with BCE in Figure 2) are shown to come from the Central Asian ancestor node 14 (Figure 2) at ca. 0.97 Ma. Therefore, Central Asia shows a remarkable relation to the origin of Thermopsideae in temporal-spatial dimensions, in other words, evolution of Thermopsideae in Central Asia, was coupling with the multiple stages of uplift of the QTP since Cenozoic, which significantly affected the paleoenvironment, paleogeography, and paleoclimate in QTP and Central Asia. To explain evolutionary process of Thermopylae using uplift is reasonable. For instance, divergence of* Piptanthus* and other taxa in the QTP and North China (node 11 in Figure 2) is dated to ca. 4 Ma, during the third phase (intensive northern QTP uplift ca. 3.6 Ma).* Piptanthus* is estimated to be young, with a diversification age of ca. 0.85 Ma, which falls into the period of QTP maximum iceosphere (cryosphere) during the third phase of uplift [66, 68]. Many people have discussed the East Asia-North America disjunction in regard to* Thermopsis* [14, 28, 51, 52, 69]. Yuan et al. [69–71] and Peng and Yuan [51] considered* Thermopsis* section* Archithemopsis,* C. J. Chen, R. Sa, and P. C. Li, to be occurring in Sino-Japanese regions, as the primitive group in the genus. Sa et al. [28] concluded that* Thermopsis* originated from the Sino-Japanese flora and then dispersed to North America via the Bering Strait. However, our analysis shows the ancestor of North American species to have come from Central Asia (node 8 in Figure 2) rather than East Asia and that the East Asia clade also on the whole evolved in Central Asia, which is different from previous hypotheses. In addition, a new genus* Vuralia* is erected from Thermopsis and related to North American node in the ITS phylogenetic tree [15], whereas* Thermopsis chinensis* and* Th. fabacea* (Figure 2) are also included in North American node; therefore, these three species in Eurasia can be regarded as the dispersals from North America and recent event in Pliocene-Pleistocene from North America crown root 5 Ma, as shown in Figures 2 and 3.

Meanwhile, our estimated diversification age of the Eurasia (Central Asia)-North America disjunction within* Thermopsis* is ca. 20.32 Ma (node 8 in Figure 2). This early Miocene time is similar to that of most other genera showing East Asia-North America disjunctions, for example,* Cercis* ca. 15.41 Ma,* Torreya* ca. 16.7 Ma [72],* Cornus* 13.1 Ma [73],* Calycanthus* 16 Ma [74],* Epimedium-Vancouveria* 9.7 Ma [75], and* Hamamelis *7.7-7.1 Ma [76], but is different from* Kelloggia* with 5.42 Ma [77] and* Phryma* 5.23-3.68 [78]. As mentioned above, another diversification between Eurasia-North America disjunction which originates from North America at ca. 5 Ma Pliocene-Pleistocene (node 9, Figure 2) resembles* Kelloggia *and* Phryma.*

From Central Asia, a western dispersal route via the Caucasus arriving at the Mediterranean, is illustrated with* Anagyris* in Figures 2 and 3. Our dating of* Anagyris* is 3.08 Ma, which is different from the suggested age of 8.2 ± 4.5 Ma [12]. The* Anagyris* estimate of Ortega-Olivencia and Catalán [12] probably lacks denser sampling from Thermopsideae, since only 5-6 species were selected in total. In general, sufficient samples are necessary in dating.* Vuvalia,* another Mediterranean genus that belongs to the North America clade (node 9 in Figure 2), as well as* Thermopsis chinensis* and* Th. fabacea,* probably dispersed from North America in Pliocene-Pleistocene, since our estimated crown age of North America clade node 9 is about 5 Ma; see Figures 2 and 3.

### 4.4. Origin and Geographic Diversification of* Ammopiptanthus*

In view of the unique evergreen broadleaf habit of* Ammopiptanthus* in the desert region of northwestern China and Kyrgyzstan, many people have speculated that* Ammopiptanthus* is a relict of the evergreen broadleaf forest of this region from the Tertiary period [14, 26–29, 79, 80]. The two species form an obvious disjunction pattern,* A. mongolicus* distributed in western Inner Mongolia and the south Gobi desert and* A. nanus* in the western Tianshan Mts. restricted to the borders between China and Kyrgyzstan [29]. Both species of* Ammopiptanthus* are diploid [81]. Genetic diversity from ISSR analysis [29] indicated that differentiation of the two species was significant. In view of its high genetic diversity, a vicariance possibly resulted from the fragmentation of the ancestor range.

From the present analysis (Figure 2),* Ammopiptanthus* is shown to be directly derived from the common ancestor of Thermopsideae, and its divergence time is estimated at ca. 28.81 Ma of middle Oligocene. As mentioned above, during this period, the climate was cooling and increasing in aridity and the vegetation was broadleaved evergreen and deciduous woodland. Therefore,* Ammopiptanthus* spatiotemporally should be speculated to be a relict survivor of the evergreen broadleaf forest at the Tertiary Oligocene.

Much evidence indicates that CO_2_ decline promoted the origin of C_4_ photosynthesis in grasses in the middle Oligocene ca. 30 Ma [82–84]. Similar to C_4_ grass plants, emergence of* Ammopiptanthus* just falls into this period, and since it favors cold and arid climates in an arid region, it can be regarded as a plant case of response to CO_2_ decline.

The vicariance and fragmentation of the two* Ammopiptanthus* species dated to ca. 3.88 lead us to link these events to the time label 3.6 Ma of QTP intense uplift and consequently to Asian interior land aridification [66, 68, 85]. This is the same as the speciation of some species in* Caragana* [86] and* Phyllolobium* [87]. The vicariance and fragmentation of the two* Ammopiptanthu*s species also likely corresponded to the low pCO_2_ and cold and arid climate that resulted from glacial intensification at late Pliocene times (~3.3 to 2.4 Ma) (another time label is at middle Miocene ~14 to 10 Ma) [88].

### 4.5. Attribute of Madrean-Tethyan Disjunction of Thermopsideae

The Madrean-Tethyan disjunction was proposed by Axelrod [89], as reviewed recently by Wen and Ickert-Bond [90], which hypothesized a nearly continuous belt of Madrean-Tethyan dry broadleaf evergreen sclerophyllous vegetation stretching from western North America to Central Asia in the early Tertiary (from Eocene to late Oligocene) at low latitudes. The former Madrean-Tethyan belt, in fact, falls into the succulent biome locating on the two sides of the Tethys in the Tertiary flora and vegetation, one of four biomes of the legume distribution pattern [21]. The representatives of taxa forming a distinctive disjunction in Thermopsideae are* Thermopsis* section* Thermia* and* Baptisia* in North America and* Thermopsis* sections excluding section* Thermia* and* Piptanthus* in Eurasia, mainly in the QTP and its adjacent regions and* Ammopiptanthus *in Central Asia and* Anagyris* in the Mediterranean. Clearly, Thermopsideae presents a Madrean-Tethyan disjunction. From the temporal dimension, our dating of Thermopsideae to Oligocene ca. 28.81 Ma, is just consistent with Axelrod [89] time range of early Tertiary Oligocene. This is different from the ages of origin of Madrean-Tethyan disjunctions (see review of Wen and Ickert-Bond [90]), for instance,* Platanus orientalis*-*P. racemosa* s.l. (Platanaceae) ca. 20.5–21.9 Ma [91];* Juniperus* was at 43.66 Ma [92]. Since Thermopsideae is illustrated to be derived from Central Asia, the evolutionary pattern of this tribe would be migration from Eurasia to North America via the Bering Strait. Moreover, types of Thermopsideae, except for members of* Thermopsis* and* Baptisia,* are perennial herbs producing rhizomes. The rest of these taxa, especially* Ammopiptanthus*, are shrubby and likely to be relicts of the Tertiary dry broadleaf evergreen sclerophyllous vegetation [89]. This, in fact, provides a relict status of dry broadleaf evergreen sclerophyllous vegetation of Madrean-Tethyan disjunction. Therefore, from perspectives of distribution, dated age, and vegetation of Thermopsideae, it fits as a good case of Madrean-Tethyan disjunction.

### 4.6. African Origin and Dispersal of Core Genistoides

The so-called core genistoides are defined on the basis of molecular phylogeny [1, 3–5, 16–19]. This clade has four tribes taxonomically [19], namely, Crotalarieae, Podalyrieae, Genisteae, and Thermopsideae. Except for Thermopsideae, the tribes occur mainly in Africa, and only a few species expand to the Mediterranean, southern Europe, the Middle East, the Caucasus, and Russia [6–8].

Schrire et al. [21] stated that the derived genistoides, including Crotalarieae, Podalyrieae, and Genisteae, have their basal branching elements in warm temperate southern Africa, an ancestral crown in the southern warm temperate biome [22]. From there, they would have migrated northwards through montane tropical Africa to the Mediterranean and Macaronesian regions and sequentially to the New World, or have secondarily invaded the tropics.

Our molecular dating and S-DIVA results (Figures 2 and 3) indicate that the core genistoides originated from Africa, probably warm temperate southern Africa as mentioned above [21], from Eocene to Oligocene ca. 54.43–33.47 Ma (Nodes 1,3). The four tribes not only dispersed to the Mediterranean, West Asia, the Caucasus, northwestern Russia, Central Asia, East Asia, and North America, but also continuously diversified in Africa in situ until middle Miocene ca. 12 Ma, which is fundamentally due to the diversification of the three tribes Crotalarieae, Podalyrieae, and Genisteae in that continent (Figure 2). The exact place of origin of these three tribes will probably become less ambiguous due to discovery of fossil records and increased taxon sampling and sequencing and so forth. However, an African origin is affirmed; furthermore, the biome warm temperate southern Africa, sensu Schrire et al. [21], is accepted here. This also illuminates the origin of Thermopsideae.

## Supplementary Material

Outgroups, include seven fossil genera, and taxa of core genistoides.

## Figures and Tables

**Table 1:** Sources of plant materials.

Species	Voucher	Source	ITS	*rps*16	*psb*A*-trn*H	*trn*L-F
*Ammopiptanthus* S.H. Cheng						
*A. mongolicus* (Maxim.) S.H. Cheng	W.J. Zhu 64004 (HNWP)	Wuda, Inner Mongolia, China		KP636600	KP636576	KP636624
*A. mongolicus* (Maxim.) S.H. Cheng	M.L. Zhang s.n. (XJBI)	Turpan Eremophytes Botanic Garden, China	KP636562			KP636625
*A. nanus* (Popov) S.H. Cheng	P. Yan, M. Ma 4280 (SHI)	Wuqia, Xinjiang, China		KP636601	KP636577	KP636626
*A. nanus* (Popov) S.H. Cheng	Y.H. Wu 870001 (HNWP)	Wuqia, Xinjiang, China				KP636627
*A. nanus* (Popov) S.H. Cheng	M.L. Zhang s.n. (XJBI)	Turpan Eremophytes Botanic Garden, China	KP636563			KP636628
*Piptanthus* Sweet						
*P. concolor* Harrow ex Craib	Tibet Medicine Exp. Team 213 (HNWP)	Jilong, Tibet, China	KP636564	KP636602	KP636578	KP636629
*P. laburnifolius* (D. Don) Stapf	Qinghai Exp. Team 750501 (HNWP)	Longzi-Zhunba, Tibet, China	KP636565	KP636603	KP636579	KP636630
*P. leiocarpus* Stapf	Tibet Medicine Exp. Team 1576 (HNWP)	Nielamu, Tibet, China	KP636566	KP636604	KP636580	
*P. nepalensis* Sweet	ITS: Wang HC, 0121 (KUN);	ITS: Yunnan, China;	AF215922		KP636581	KP636631
	Ciduo Cidan, et al. 2436 (PE)	Yadong, Tibet, China				
*Thermopsis* R. Br.						
*T. alpina* Ledeb.	ITS: Saren 2000;	ITS: Tibet, China;	AF123447	KP636605	KP636582	KP636632
	Y.H. Wu 29102 (HNWP)	Maduo, Qinghai, China				
*T. alpina* Ledeb.	Y.H. Wu 28862 (HNWP)	Yushu, Qinghai, China		KP636606		KP636633
*T. alpina* Ledeb.	P. Yan, J.Y. Guo 6790 (SHI)	Manasi, Xinjiang, China		KP636607	KP636583	KP636634
*T. alpina* Ledeb.	Pamier Exp. Team 5233 (SHI)	Tashikuergan, Xinjiang, China	KP636567	KP636608	KP636584	KP636635
*T. alpina* Ledeb.	J. Tao, et al. 1067 (SHI)	Manasi, Xinjiang, China			KP636585	KP636636
*T. barbata* Benth.	Tibet Medicine Exp. Team 4329 (HNWP)	Jiacha, Tibet, China	KP636568	KP636609		KP636637
*T. inflata* Cambess.	ITS: Liu JQ s.n.;	ITS: Qinghai, China;	AF123451	KP636610	KP636586	KP636638
	Z.Y. Wu, S.K. Chen, Q. Du 75–166 (HNWP)	Bogu lake-Malashan, Tibet, China				
*T. inflata* Cambess.	XJBI Tibet Team s.n. (XJBI)	Zada, Tibet, China		KP636611		KP636639
*T. inflata* Cambess.	XJBI Tibet Team s.n. (XJBI)	Gaize, Tibet, China		KP636612	KP636587	KP636640
*T. kaxgarica* Ch.Y. Yang	C. Yan s.n. (XJBI)	Turpan, Xinjiang, China		KP636613	KP636588	KP636641
*T. lanceolata* R. Br.	ITS: Saren 010 (PE);	ITS: Qinghai, China;	AF123448	KP636614	KP636589	KP636642
	Y.H. Wu 36480 (HNWP)	Dulan, Qinghai, China				
*T. lanceolata* R. Br.	XJBI Exp. Team s.n. (XJBI)	Sawuershan, Xinjiang, China		KP636615	KP636590	KP636643
*T. licentiana* E. Peter	R.F. Huang 2677 (HNWP)	Tianzhu, Gansu, China	KP636569	KP636616	KP636591	KP636644
*T. licentiana* E. Peter	Y.H. Wu 1014 (HNWP)	Yecheng, Xinjiang, China		KP636617		
*T. licentiana* E. Peter	Guoluo Exp. Team 649 (HNWP)	Jiuzhi, Qinghai, China			KP636592	KP636645
*T. licentiana* E. Peter	H.B.G. 198 (HNWP)	Maxin, Qinghai, China			KP636593	KP636646
*T. mongolica* Czefr.	P. Yan 3368 (SHI)	Hefeng, Xinjiang, China	KP636570	KP636618	KP636594	KP636647
*T. przewalskii* Czefr.	B.Z. Guo 10267 (HNWP)	Tongren, Qinghai, China	KP636571	KP636619	KP636595	KP636648
*T. schischkinii* Czefr.	S.M. Duan s.n. (XJBI)	Wuma, Tibet, China	KP636572	KP636620	KP636596	KP636649
*T. smithiana* E. Peter	XJBI Exp. Team s.n. (XJBI)	Geji, Tibet, China	KP636573	KP636621	KP636597	KP636650
*T. turkestanica* (Gand.) Ch.Y. Yang	Tibet-Xinjiang Exp. Team 1044 (HNWP)	Zhaosu, Xinjiang, China	KP636574	KP636622	KP636598	KP636651
*T. yushuensis* S.Q. Wei	S.W. Liu 609 (HNWP)	Yushu, Qinghai, China	KP636575	KP636623	KP636599	KP636652

HNWP (Herbarium, Northwest Institute of Plateau Biology, Chinese Academy of Sciences, Xining, Qinghai); PE (China National Herbarium, Institute of Botany, Chinese Academy of Sciences, Beijing); SHI (Herbarium, Shihezi University, Shihezi, Xinjiang); and XJBI (Xinjiang Institute of Ecology and Geography, Chinese Academy of Sciences, Urumqi, Xinjiang).

**Table 2:** References for fossils of seven genera used to constrain ages for dating.

Taxa	Time (Ma)	Location	Reference
*Cercis *	60–11 Late Cretaceous-Miocene	China	Tao, 1992 [58]; Tao et al., 2000 [42]
*Cercis *	Eocene	N America	Lavin et al., 2005 [22]
*Acacia *	47–42 Eocene	Liaoning, China; Tanzania	Tao et al., 2000 [42]; Lavin et al., 2005 [22]
*Acacia *	15 Miocene	Dominican Rep.	Lavin et al., 2005 [22]
*Bauhinia *	ca. 65 later Cretaceous	Helongjiang, China,	Tao et al., 2000 [42]
*Cladrastis *	40–20 middle Eocene	Tennessee, N America	Herendeen et al., 1992 [20]
*Cladrastis *	Miocene	Inner Mongolia	Tao et al., 2000 [42]
*Sophora *	35–9 Oligocene-Miocene	China, Siberia, N America	Tao et al., 2000 [42]; IB & NIGP, 1978 [43]
*Pueraria *	17–5 Miocene	Yunnan, Shandong, China	Tao, 1992 [58]; Tao et al., 2000 [42]
*Dalbergia *	19.5–5 Miocene	China, N America, Europe	IB & NIGP, 1978 [43]; Tao, 1992 [58]; Tao et al., 2000 [42]

IB and NIGP: Institute of Botany and Nanjing Institute of Geology and Palaeontology, Academia Sinica.
