# Supplementary material for: Molecular Biogeography of Tribe Thermopsideae (Leguminosae): A Madrean-Tethyan Disjunction Pattern with an African Origin of Core Genistoides
Source: Biomed Res Int. 2015 May 31;2015:864804. doi: 10.1155/2015/864804 (PMC4465657; doi:10.1155/2015/864804)
Supplement: Supplementary file 1 — Outgroups, include seven fossil genera, and taxa of core genistoides. [file 864804.f1.pdf]

S 1. Outgroups, including seven species, were downloaded from GenBank for Fig. 1.

ITS: *Sophora davidii* (Franch.) Skeels AF467496; *S. flavescens* Aiton AF123452; *S. microphylla* Aiton AY056075; *Podalyria sericea* (Andrews) R. Br. ex Ait. DQ289611; *Cytisus balansae* (Boiss.) Ball DQ289688; *C. maurus* Humbert & Maire AF351118; *C. oromediterraneus* Rivas Mart., T.E. Díaz, Fern. Prieto, Loidi & Penas AF351108;

*psb*A-*trn*H: *Sophora davidii* (Franch.) Skeels JF725696; *S. flavescens* Aiton JF725695; *S. microphylla* Aiton GQ248391;

*trn*L-F: *Sophora davidii* (Franch.) Skeels JF725665; *S. flavescens* Aiton JF725666; *Podalyria sericea* (Andrews) R. Br. ex Ait. DQ289764; *Cytisus balansae* (Boiss.) Ball DG289789; *C. maurus* Humbert & Maire AF352213; *C. oromediterraneus* Rivas Mart., T.E. Díaz, Fern. Prieto, Loidi & Penas GQ289768.

S 2. Outgroups, including seven fossil genera, and taxa of core genistoides not appearing in Table 1, following ITS sequence data were downloaded from GenBank for Fig. 2.

Seven fossil genera:

Acacia farnesiana Wall. AF360728; *A. mearnsi* De Will. AF360750; *A. senegal* (L.) Willd AF360727;

Cercis canadensis L. AF390188; *C. chinensis* Bunge AF286351; *C. chingii* Chun AF286350; *C. chuniana* Metcalf AF286349; *C. gigantea* Cheng and Keng f. AF390194; *C. occidentalis* Torr. AF286352; *C. racemosa* Oliver AF288782; *C. Siliquastrum* L. AF286353;

Bauhinia guianensis var. kunthiana (Vogel) Wunderlin FJ037825;

Dalbergia congestiflora Pittier AF068140; *D. foliolosa* Benth. AF189002; *D. sissoo* Roxb. AF189023;

Pueraria lobata (Willd.) Ohwi (= *P. montana* var. *lobata*) AF338215; *P. montana* (Lour.) Merr. AF338216; *P. thomsonii* Benth. (= *P. montana* var. *thomsonii*) AF338217;

Sophora affinis Torr. et Gary U59886; *S. chrysophylla* Seem. AY056070; *S.*

microphylla Ait. var. *longicarinata* AY056074; *S. secundiflora* Laq. ex DC. AF174638;

Pueraria lobata (Willd.) Ohwi (= *P. montana* var. *lobata*) AF338215; *P. montana* (Lour.) Merr. AF338216; *P. thomsonii* Benth. (= *P. montana* var. *thomsonii*) AF338217;

Cladrastis delevayi Prain EF457712; *C. platycarpa* Makino EF457713;

Cyclopia alpina A.L.Schutte AM050830; *C. aurescens* Kies AM050826; *C. burtonii* Hofmeyr & E.Phillips AM050823; *C. galioides* DC. AM050825

Amphithalea alba R.Granby AM261217; *A. axillaris* R.Granby AM261218;

Stirtonanthus taylorianus (L.Bolus) B.-E. Van Wyk & A.L.Schuttea AJ409907; *S. insignis* (Compton) B.-E. Van Wyk & A.L.Schutte AJ409906;

Podalyria calyptrata Willd. AF287670; *P. speciosa* Eckl. & Zeyh. AF287671;

Liparia umbellifera Thunb. AF287665; *L. vestita* Thunb. AM261492;

Virgilia divaricata Adamson AJ409910;

Calpurnia intrusa E.Mey. AF287668; *C. aurea* Baker CAU59887;

Robynsiophyton vanderystii R.Wilczek EU347878;

Spartidium saharae Pomel EF457729;

Lebeckia cytisoides Thunb.EF457719; *L. wrightii* Bolus EF457699;

Wiborgia obcordata Thunb.EF457697; *W. mucronata* Druce EF457696;

Rafnia capensis Druce EF457702; *R.* amplexicaulis Thunb.EF457701;

Aspalathus venosa E.Mey.EU000615; *A. tuberculata* Walp. EU000614;

Lotononis laxa Eckl. & Zeyh.AF287677; *L. lotononoides* (Scott-Elliot) B.-E. Van Wyk AF287676;

Bolusia amboensis Harms EU347891;

Crotalaria pallida Aiton GQ470553; *C. incana* L. GQ470552;

Pearsonia sessilifolia Dümmer AF287675; *P. cajanifolia* (Harv.) Polhill EU347876;

Rothia hirsuta Baker EU347877;

Xiphotheca cordifolia A.L.Schutte & B.-E.van Wyk AM261679; *X. elliptica* (DC.) A.L.Schutte & B.-E.van Wyk AM261680;

Echinospartum barnadesii Fourr. FJ897733;

Ulex micranthus Lange AY263687; *U. parviflorus* Pourr. AF443626;

Lupinus affinis var. carnosulus (Greene) Jeps. AF007487; *L. albescens* DQ524190;

Retama monosperma (L.) Boiss. DQ524326; *R. sphaerocarpa* Boiss. AY263683;

Genista florida Asso AF351087; *G. pumila* (Debeaux ex Hervier) Vierh. subsp. *eliassennenii* (Uribe-Ech. & Urrutia) Rivas Mart., Fern.Gonz., Sánchez Mata & J.M.Pizarro AY263658; *G. cinerea* (Vill.) DC. *cinerea* AY263636;

Cytisus grandiflorus DC. subsp. *haplophyllus* AF352212; *C. villosus* C.Presl ZT0087;

Cyclopia glabra (Hofmeyr & E. Phillips) A.L.Schutte AF287666;

Podalyria calyptrata (Retz.) Willd. AF287670; *P. speciosa* Eckl. & Zeyh AF287671;

Ammopiptanthus mongolicus (Maxim. ex Kom.) Cheng f. AF209784; *A. nanus* (M. Pop.) Cheng f. AY091567;

Amphithalea ericifolia (L.) Eckl & Zeyh AF287673;

Anagyris foetida L. AY091571; *A. latifolia* Brouss. ex Willd. FJ482249; *A.* latifolia Brouss. ex Willd FJ482248; *A.* latifolia Brouss. ex Willd FJ482247;

Baptisia alba (L.) Vent. AY773348; *B. australis* (L.) R. Br. AY091572; *B. bracteata* Muhl. ex Ell. AY773349; *B. cinerea* (Raf.) Fern. & Schub. AY773350; *B. sphaerocarpa* Nutt. AY773351;

Piptanthus leiocarpus Stapf AY091569; *P. tomentosus* Franch AY091570;

Thermopsis alpina (Pallas) Ledeb. AF123447; *T. barbata* Benth AY773353; *T. chinensis* Benth. ex Moore AF123443; *T. divaricarpa* Nelson AY091575; *T. fabacea* (Pall.) DC. AY091573; *T. inflata* Camb AF123451; *T. lanceolata* R. Br. AF123448; *T. licentiana* Pet.-Stib. AF123449; *T. macrophylla* Hook. ex Arn. AF123450; *T. montana* Nutt. ex Torrey & A. Gray AY091574; *T. rhombifolia* (Nutt. ex Pursh) Richardson AF007468; *T. smithiana* Peter-Stibal AY773354; *T. turkestanica* Gand. AF123446.
